# Spatially offset optical coherence tomography: Leveraging multiple scattering for high-contrast imaging at depth in turbid media

**DOI:** 10.1126/sciadv.adh5435

**Published:** 2023-07-07

**Authors:** Gavrielle R. Untracht, Mingzhou Chen, Philip Wijesinghe, Josep Mas, Harold T. Yura, Dominik Marti, Peter E. Andersen, Kishan Dholakia

**Affiliations:** ^1^ Department of Health Technology, Technical University of Denmark, Kongens Lyngby 2800, Denmark.; ^2^ SUPA, School of Physics and Astronomy, University of St Andrews, St Andrews KY16 9SS, UK.; ^3^ Electronics and Photonics Laboratory, The Aerospace Corporation, El Segundo, CA 90245, USA.; ^4^ Centre of Light for Life and School of Biological Sciences, University of Adelaide, Adelaide, SA 5005, Australia.

## Abstract

The penetration depth of optical coherence tomography (OCT) reaches well beyond conventional microscopy; however, signal reduction with depth leads to rapid degradation of the signal below the noise level. The pursuit of imaging at depth has been largely approached by extinguishing multiple scattering. However, in OCT, multiple scattering substantially contributes to image formation at depth. Here, we investigate the role of multiple scattering in OCT image contrast and postulate that, in OCT, multiple scattering can enhance image contrast at depth. We introduce an original geometry that completely decouples the incident and collection fields by introducing a spatial offset between them, leading to preferential collection of multiply scattered light. A wave optics–based theoretical framework supports our experimentally demonstrated improvement in contrast. The effective signal attenuation can be reduced by more than 24 decibels. Notably, a ninefold enhancement in image contrast at depth is observed in scattering biological samples. This geometry enables a powerful capacity to dynamically tune for contrast at depth.

## Teaser

Selective collection of multiply scattered light leads to better image contrast at depth in optical coherence tomography.

## INTRODUCTION

Morphological imaging with optical coherence tomography (OCT) has made major strides in the past few decades (*1*, *2*), making rapid advances into the field of retinal imaging, revolutionizing applications in ophthalmology (*3*), and expanding into other fields including dermatology (*4*), cardiology (*5*), and the early detection of cancer (*6*). It is also used in numerous areas outside of biomedicine, such as art conservation (*7*) and nondestructive testing (*8*). OCT relies on the coherence of light backscattered from the sample, with interference playing the important role of gating, allowing an accurate determination of the spatial origin of the retrieved signal. Backscattering occurs when there is a refractive index mismatch, typically generated by subresolution scatterers present within the sample microstructure. While advances in OCT have been spectacular, there are major drawbacks in terms of its resolving capacity at depth. The state-of-the-art implementations of OCT use light in the near-infrared range of the spectrum to penetrate deeper into biological matter (*9*); however, signal extinction through turbid samples often precludes obtaining discernible signals from depths beyond 1 mm. The conventional wisdom is that the OCT signal is dominated by ballistically scattered light (light that has undergone a single backscattering event), whereas multiply scattered light and diffuse light are detrimental to image formation. In this study, we present and demonstrate an alternate viewpoint that selective collection of multiply scattered light can lead to improved image contrast at depth in OCT, particularly in highly scattering samples.

Light propagation in disordered media has come to the fore as a fundamental problem for all imaging, including deep tissue imaging with OCT. Strongly scattering media lead to multiple scattering of the incident field, which induces aberrations, generates a diffuse background haze in images, and precludes extraction of information from depth. While previous works have acknowledged that multiple scattering plays a role in OCT (*10*, *11*), most advances in imaging through disordered media have focused on the enhancement of the single-to-multiple scattering ratio (SMR) (*12*). For instance, adaptive optics approaches to correcting wavefront aberrations using a spatial light modulator have increased the depth obtained (*12*, *13*). Alternatively, a dual-axis geometry with overlapping beams has been developed to reduce multiple scattering in both confocal microscopy (*14*) and dual-axis OCT (*15*, *16*). Dark-field approaches, including in OCT (*17*), have also been used to reject extraneous background noise, such as from reflective imaging interfaces. These results have shown promise in breaching the fundamental limit in depth penetration and greatly enhancing image contrast in microscopy. However, in OCT, these approaches have failed to consider the accompanying limits in the dynamic range and the detection noise. Typically, OCT can gather information at depths of 10 times the scattering mean free path of the media (*12*), which is determined by the capacity of the coherence gate to reject the temporally delayed signal from multiple scattering. Because of scattering in the illumination and detection paths, past 10 scattering lengths, we can expect an extinction of more than 100 dB in the detectable signal. This value is commensurate with the sensitivity of conventional OCT systems, i.e., where a 100-dB attenuated reflector is still detectable above the noise floor. Furthermore, quantization of the acquired spectrum often limits the effective dynamic range to 40 to 60 dB (*18*). In most practical settings, the high flux from surface-scattered photons will obscure depth information within the detector noise before the fundamental limit in SMR is reached. Previous demonstrations in extending depth penetration through the reduction of multiple scattering have shown the identification of highly reflective or scattering samples below aberrating media (*12*, *16*, *19*). However, their utility in the practical cases of uniformly scattering biological tissue is yet to be determined.

While this theory suggests that OCT cannot image past approximately 10 scattering lengths based on dynamic range alone, which is commensurate with confocal microscopy (*12*), practically, OCT is well known to retrieve morphological tissue contrast at depths much greater than most microscopy methods. This is because a large proportion of the useful OCT signal also comprises multiply scattered light (*10*, *20*). In uniformly scattering tissues, multiple scattering dominates the signal past 1 mean free path in the sample. Despite the aversion to multiple scattering in microscopy, much of this multiple scattering leads to temporal delays that are lower than the coherence time of the source and, thus, still contribute to the well-discernible morphological contrast (*20*). While ballistic scattering attenuates exponentially, the likelihood of multiple scattering increases with depth.

**Fig. 1.**
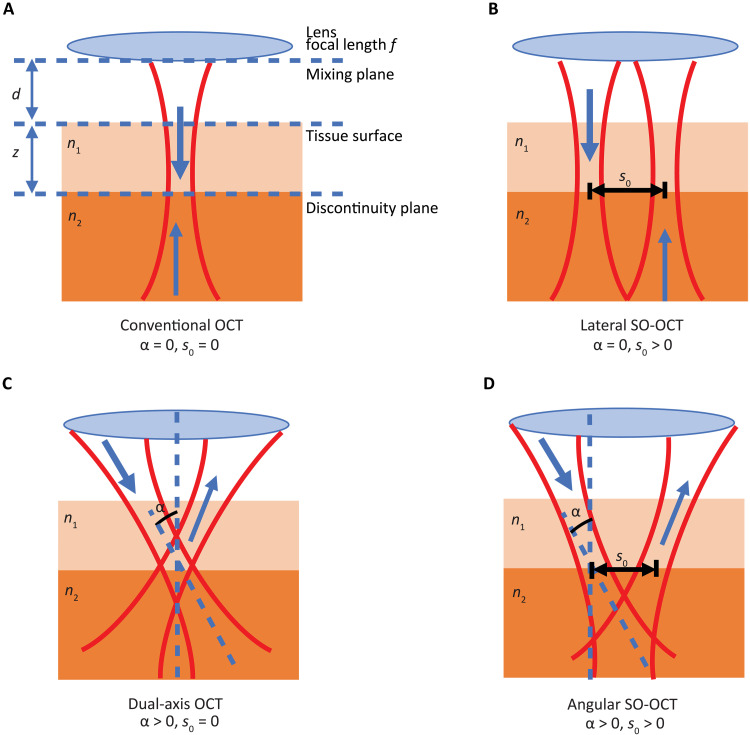
A comparison of the sample arm geometry for conventional OCT, SO-OCT, and other offset geometries. (**A**) Conventional OCT geometry. (**B**) SO-OCT, as proposed here, comprises an offset introduced between parallel illumination and collection paths. (**C**) Dual-axis OCT, with an angular offset between the illumination and collection paths and a region of overlap. Dark-field OCT also comprises an angular offset between the illumination and collection path but uses annular, off-axis illumination and collection through the center of the lens. (**D**) Angular SO-OCT, which comprises simultaneous dual-axis OCT and SO-OCT. *f*, lens focal length; *d*, distance from the lens to the tissue surface; *n*_1_ and *n*_2_, refractive index of two tissue layers; *s*_0_, lateral offset between the illumination and collection paths in the discontinuity plane where the depth-dependent offset *s*(*z*) = *z* tan α + *s*_0_; α, half-angular offset between the illumination and collection path, measured relative to a normal to the tissue surface. In the case of SO-OCT, α = 0 and *s*(*z*) = *s*_0_.

Recognizing this phenomenon, it becomes pertinent to investigate the information content of the multiply scattered signal and whether the multiply scattered signal alone can be used to form an image from deeper in the sample. Previous studies have alluded that the proportion of reflected, ballistic, and multiply scattered light could be varied by modifying the relative geometries of the illumination and collection paths. For instance, dark-field OCT (*17*) rejected on-axis reflections from imaging interfaces to enhance contrast in high-resolution cell imaging, while dual-axis OCT (*15*) sought to enhance single scattering at depth precisely at the focus of the intersection of tilted illumination and detection paths. Here, we propose an experimental concept to investigate the role of multiple scattering in OCT image contrast. As a counterpoint to all previous studies, we illustrate that we can generate an OCT image by prioritizing the multiple scattering signal. By completely decoupling the incident and collection paths of OCT though a facile spatial offset, we can tune the collection of ballistic light and multiply scattered light at different depths in the sample. This leads to a substantially enhanced contrast at depth by counteracting the attenuation in scattering samples and preserving the signal within the dynamic range of the detector. We term our technique spatially offset OCT (SO-OCT) (*21*). An overview of the sample arm geometry for SO-OCT relative to conventional OCT is shown in Fig. 1 (A and B).

**Fig. 2.**
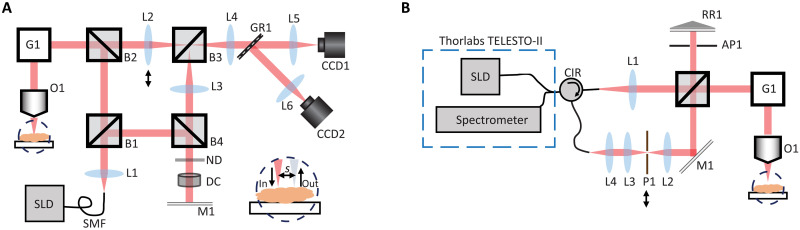
Experimental diagrams of SO-OCT systems. (**A**) Experimental diagram of the homebuilt SO-OCT system at 800 nm. The inset at the bottom right shows the offset between the laser and collection point due to the shift of lens L2. The double arrow indicates the direction of translation of lens L2. (**B**) Experimental diagram of the SO-OCT system built as an add-on to a commercial instrument at 1295 nm. Offset is introduced by translating the pinhole in the collection path (P1) as indicated by the arrow in the diagram. SLD, superluminescent diode; SMF, single-mode fiber; O1, microscope objective; G1, two-dimensional galvo mirror; B1, nonpolarizing beamsplitter cube [BS029, Thorlabs, 90:10 (reflection:transmission)]; B2 to B4, nonpolarizing beamsplitter cubes [BS014, Thorlabs, 50:50 (R:T)]; L1 to L6, lens; ND, neutral density filter; DC, dispersion compensation; M1, mirror; CCD1, charge-coupled device (CCD) camera (FL3-U3-32S2M-CS, Point Grey); CCD2, GigE Vision line scan camera (Aviiva EM1, Teledyne); GR1, a transmission grating (1200 lines/mm, coated for 700 to 960 nm); CIR, circulator; RR1, retroreflector; AP1, variable aperture.

Here, we present both an experimental demonstration of SO-OCT and a theoretical framework that provides a unified approach for exploring the impact of offset geometries in OCT, including SO-OCT, dual-axis OCT (*15*), and dark-field OCT (*17*). A wave-based model of the heterodyne efficiency factor (*10*, *22*) provides a framework for exploring the impact of introducing various offset collection geometries. While others have explored similar collection geometries (*15*, *17*, *23*, *24*), none have yet adequately described the physics that underpins this collection of techniques (as outlined in Fig. 1). We show that we can experimentally separate the ballistic and multiply scattered components of the measured OCT signal by tuning the offset. Subsequently, we show that selective collection of the multiply scattered signal leads to substantially enhanced contrast at depth and reveals mesoscale features obscured by the limits of detection noise despite favoring multiple scattering. Furthermore, our offset geometry adds additional contrast from collecting a varied proportion of scattering angles from the sample. We validate SO-OCT on two frequency domain OCT systems with different central wavelengths and configurations using phantoms, and we demonstrate its effectiveness on biological samples with a range of scattering properties. The first experimental setup (Fig. 2A) is homebuilt and requires the translation of a single optical component to achieve enhancement on both image quality and depth penetration. The second experimental setup (Fig. 2B) is an adaptation of a commercially available frequency domain OCT system with a custom-built add-on enabling spatially offset detection, which may be readily incorporated into many existing OCT systems. These two systems are described in detail in Materials and Methods. Through the use of these two systems, we validate that SO-OCT is applicable over a broad parameter space where OCT is used and that the selection of the optimal wavelength can be based on the sample properties similarly to conventional OCT. Samples were selected on the basis of the optical properties to highlight the imaging capabilities of each system. Our approach reveals that improving penetration depth should not be addressed solely from the perspective of eliminating multiple scattering. On the contrary, SO-OCT demonstrates an approach for capturing information about the sample that is carried predominantly by the multiply scattered light.

## RESULTS

### Comprehensive theoretical framework and modeling

The physical principle of SO-OCT is detailed in Materials and Methods. Briefly, light backscattered from the sample can be classified into three groups: ballistic light, which is directly collected after a single scattering event; multiply scattered light, which contains information about the sample and undergoes a few scattering events while still returning to the detector within the coherence gate; and diffusely scattered light, which presents as a diffuse haze and degrades image quality and resolution (*25*). Our expanded modeling based on the extended Huygens-Fresnel (EHF) principle (*10*) shows that we can separate the multiply scattered signal by introducing a spatial offset between the illumination and collection paths. SO-OCT leads to the preferential collection of the second group (multiply scattered light) and the integration of a broader angle of the scattering phase function (*26*) in depth. These factors culminate in the compensation of the effective attenuation profile seen in the reconstructed images and maintains signal above the noise floor of detection.

We have derived a unified theoretical framework that allows us to explore the role of multiple scattering and spatial offset on the collected OCT signal. The full derivation is presented in the Supplementary Materials, but we include the final expression here for reference. When there is a separation *s* introduced between the illumination and collection paths, which, for now, are assumed to be parallel, we can write an expression for the heterodyne efficiency factor of the OCT system as

$$\text{Ψ}(z,s)=e^{-2\mu_{a}z}\left[e^{-2\mu_{s}z}e^{\frac{-s^{2}}{2\omega_{H}^{2}}}+\frac{4e^{-\mu_{s}z}(1-e^{-\mu_{s}z})}{(1+\mu_{a}\text{Δ}z_{D})\left(1+\frac{\omega_{\mathrm{SA}}^{2}}{\omega_{H}^{2}}\right)}e^{\frac{-s^{2}}{\omega_{H}^{2}+\omega_{\mathrm{SA}}^{2}}}+\frac{{\left(1-e^{-\mu_{s}z}\right)}^{2}\omega_{H}^{2}}{(1+\mu_{a}\text{Δ}z_{D})\omega_{\mathrm{SA}}^{2}}e^{\frac{-s^{2}}{2\omega_{\mathrm{SA}}^{2}}}\right]$$ (1)

where μ_s_ and μ_a_ are the scattering and absorption coefficients of the tissue, respectively, and ω_H_ and ω_SA_ are the 1/*e* intensity radii in the absence and presence of absorption and scattering, respectively. We note that this framework can readily be extended to investigate an angular offset as proposed by Zhao *et al.* (*15*) (see the Supplementary Materials). The first term on the right side of Eq. 1, which is the single scattering component (Beer’s law), is the contribution due to ballistically scattered light in the OCT signal measured in the detector plane. In OCT, ballistic light travels through the intervening turbid medium to and back from the tissue discontinuity in straight lines (i.e., without scattering). The third term represents the contribution due to multiply scattered light within the coherence time of the laser source, while the second term is a cross term related to the contribution due to both ballistic and multiply scattered return light. Note that the calculations here all consider an OCT system with dynamic focusing.

**Fig. 3.**
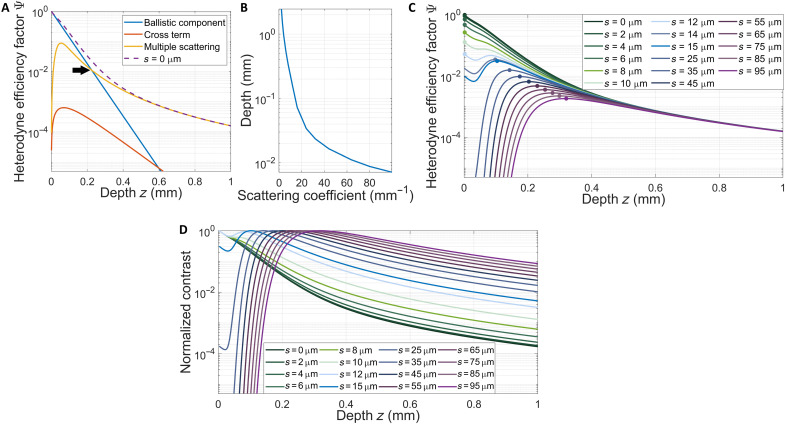
Calculations of heterodyne efficiency factor (Ψ) and normalized OCT contrast in conventional and SO-OCT. Here, the normalized OCT contrast is represented by the normalized heterodyne efficiency factor. (**A**) Relative contribution of ballistically scattered and multiply scattered light in image formation in conventional OCT. While the ballistic signal dominates superficially, the multiply scattered component rapidly dominates the signal at depth. The depth where multiple scattering begins to dominate is the crossing point between the yellow and blue lines in (A), indicated by the arrow. (**B**) Depth at which the multiply scattered light begins to dominate the OCT signal for various scattering coefficients. (**C**) Heterodyne efficiency factor in SO-OCT with various offsets. The circles plotted on each line indicate the depth with maximum contrast. (**D**) Normalized OCT contrast in SO-OCT with various offsets. The following system parameters representing the system described in Fig. 2B were used for these calculations: λ_0_ = 1295 nm; ω_0_ = 1.5 mm; *f* = 36 mm; *n* = 1.4; μ_s_ = 10 mm^−1^; μ_a_ = 0 mm^−1^; θ_rms_ = 0.3. All angles are in units of radians. Note that the calculations here all consider an OCT system with dynamic focusing.

Figure 3A indicates the relative contribution of single and multiply scattered light to the OCT signal with a separation of *s* = 0. Already from Fig. 3A, it is quite clear that multiply scattered light dominates the OCT signal acquired from deeper in the sample. We define the depth where multiply scattered light begins to dominate the OCT signal as the depth where the multiply scattered component overtakes the ballistic component and comprises a larger proportion of the total OCT signal, as indicated by the crossing point in Fig. 3A. The OCT signal is mainly composed of multiply scattered light, especially as the depth in the sample increases, contrary to the generally accepted premise that only ballistically scattered light contributes to image formation. The depth at which the multiply scattered light dominates the OCT signal depends on the scattering coefficient, as shown in Fig. 3B. As a consequence, variation of the offset, *s*, between the illumination and collection paths allows for tuning of the relative contribution of single and multiply scattered light to the collected signal. An example of the heterodyne efficiency factor for various values of *s* is shown in Fig. 3C. At very short depths for ω_H_ < *s*, the heterodyne efficiency factor is highly suppressed, as expected, because the ballistic contribution to the OCT signal is proportional to $e^{\frac{-s^{2}}{2\omega_{H}^{2}}}$. In contrast, for ω_H_ > *s*, the heterodyne efficiency factor is closely approximated for that of *s *= 0. As indicated in Fig. 3C for increasing depth *z*, Ψ coalesces with the (undisplaced) heterodyne efficiency for *s *= 0 in the regime where multiply scattered light whose temporal dispersion is less than the coherence time of the laser source are the primary contribution to the OCT signal. Practically, this demonstrates that the optimization of *s* can lead to an optimal contrast-to-noise ratio (CNR) for a selected depth.

We acknowledge that using a spatial offset filters out the ballistic component, which leads to less light being collected for higher offset (signal loss) (*10*). However, the specular reflections near the surface are also heavily suppressed. In the case where sensitivity is not a limiting factor, the dynamic range of the detector can be likewise optimized to a range relevant to the multiply scattered signals that have traveled deeper into the sample. This is illustrated in Fig. 3D, wherein the heterodyne efficiency factor has been normalized to the peak efficiency.

The optimal offset must be determined on the basis of the specific geometry and scattering properties of the sample. Because the offset can easily be tuned from *s* = 0 to various offsets with *s >* 0, images with and without the ballistic component can be acquired using the same OCT system.

### Attenuation reduction in nescofilm and cellophane tape phantoms

**Fig. 4.**
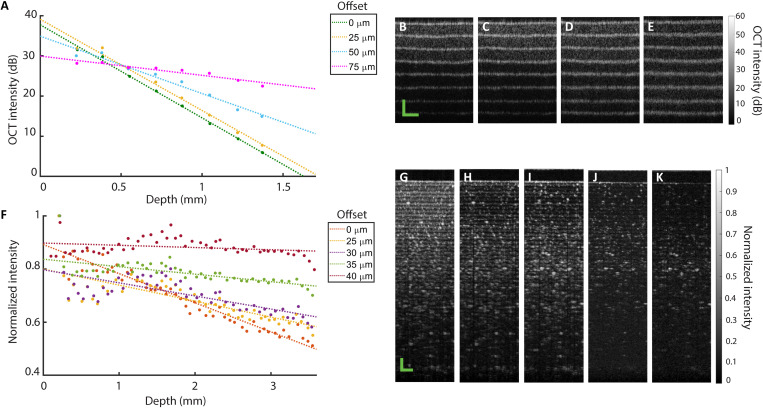
SO-OCT demonstrates reduced attenuation with increasing offset in layered phantoms. (**A**) Averaged OCT intensity across A-scans at the layer-layer interface as a function of optical depth in a seven-layer nescofilm phantom imaged at different spatial offsets. Dashed lines represent a linear fit to the log intensity, and the corresponding number is a rough approximation of the effective attenuation coefficient (per millimeters). (**B** to **E**) Corresponding B-scans for offsets of *s* = 0, 25, 50, and 75 μm, respectively. Images were acquired using the OCT system centered at 800 nm. (**F**) Averaged OCT intensity across A-scans at the layer-layer interface as a function of optical depth in a multilayer cellophane tape phantom. Dashed lines represent a linear fit to the log intensity, and the corresponding number is a rough approximation of the normalized attenuation coefficient (per millimeters). (**G** to **K**) Corresponding B-scans for offsets of *s* = 0, 25, 30, and 35 μm, 40 μm, respectively. Images were acquired using the OCT system centered at 1295 nm. Scale bars, 200 μm.

To demonstrate this in practice, we have imaged layered phantoms with both OCT systems. First, we imaged a phantom comprising seven layers of nescofilm (biological sealing film) (120 μm in thickness for one single layer) using the OCT system centered at 800 nm described in Fig. 2A. By translating the lens L2, B-scans were acquired over a range of ~1 mm with varying offsets: *s* = 0, 25, 50, and 75 μm. The exposure time of the camera (charge-coupled device) was tuned to achieve the same intensity of the reconstructed OCT signal at the first interface for fair comparison. For the offsets above, the exposure time was 30, 48, 250, and 1600 μs, respectively. Figure 4A shows the mean with respect to the lateral direction of the averaged OCT intensity at each interface imaged at varying offsets. The corresponding B-scans are visualized in Fig. 4 (B to E). We can clearly observe a reduction in the total effective attenuation as the offset is increased, which leads to an evident improvement in the layer-layer interface contrast with depth.

Similarly, we imaged a phantom comprising many layers of cellophane tape using the OCT system centered at 1295 nm and described in Fig. 2B. More layers were required because the 1295-nm system inherently has a larger imaging range as a consequence of the higher spectrometer resolution and the longer central wavelength. By translating the pinhole in the collection path, B-scans were acquired over a range of ~1 mm with varying offsets *s* = 0, 25, 30, 35, and 40 μm. Because this system is based on a modification of a commercial system and operated through software provided by the manufacturer, the exposure time could not be freely adjusted, and we were unable to achieve the same sensitivity at the surface in all images. All images were acquired in high sensitivity mode with an exposure time of approximately 180 μs. Instead, the images are normalized to the surface intensity. This limits the range of accessible offsets due to the simultaneous enhancement of the signal and noise floor during normalization. We note that the optimal offset for a particular sample will depend on the scattering properties; for some samples, we may not have been able to achieve the optimal offset with this system. Figure 4F shows the mean with respect to the lateral direction of the averaged OCT intensity at each interface imaged at varying offsets. The corresponding B-scans are visualized in Fig. 4 (G to K).

In low-scattering media or at shallow depths (i.e., within a few mean free path lengths), the well-known Beer-Lambert exponential function is a reasonable approximation of the attenuation of the incident beam, which is reflected by the linear decay of the log intensity when the spatial offset *s* = 0. We can estimate the mean free path *l*_s_ of nescofilm as 180 μm by fitting the Beer-Lambert law to the intensity attenuation in standard OCT images and finding the depth where the signal drops below 1/*e* of the maximum value. However, the probability of the number of scattering events comprising the local OCT signal increases with depth, and so does the heterodyne detection efficiency of the multiply scattered light (Fig. 3A) (*10*). As a consequence, in SO-OCT, attenuation is counteracted because the offset ensures a preferential collection of light that has undergone a few scattering events. From the linear gradient of intensities, the effective OCT attenuation coefficient can be approximated as 5.5, 5.2, 3.5, and 1.2 mm^−1^ for *s* = 0, 25, 50, and 75 μm, respectively. From Fig. 4, we see around a 4.6-fold decrease in attenuation at *s* = 75 μm. A similar analysis can be performed on the cellophane tape data where the normalized attenuation rate is correlated with the OCT attenuation coefficient. The normalized attenuation rate can be approximated as 0.11, 0.06, 0.05, 0.03, and 0.01 mm^−1^ for *s* = 0, 25, 30, 35, and 40 μm, respectively.

The effects of multiple scattering, relevant to turbid media, i.e., most biological tissues of interest, in OCT have been investigated in great detail (*20*). For mainly forward scattering samples, the temporally dispersive effect of multiple scattering may be negligible over few millimeters. We can observe this via the broadening in the thickness of the nescofilm layer interface. At 1-mm depth, the broadening of the layer interface thickness was 2.9, 23.5, and 47.0% for 25-, 50-, and 75-μm offsets, respectively, which represents a trade-off between attenuation and resolution. A similar broadening effect is visible in the thickness of the cellophane tape interfaces.

### Enhanced contrast in microbead phantoms

The offset in collection makes SO-OCT more sensitive to multiply scattered light and the angle-dependent scattering properties of the sample. We can consider each detection event in SO-OCT to comprise a few forward scattering events that introduce temporal dispersion on the order of less than the coherence time of OCT. The probability of backscattering at a given angle is determined by the scattering phase function. For sample scatterers of diameter *d* << λ, this scattering can be described by Rayleigh scattering, which has a substantial chance to scatter in all directions; scatterers of *d* < λ can be described by Mie scattering, with predominantly forward and backscattering as *d* → λ; and scatterers *d* > λ are described by geometrical optics, which backscatter with a broad range of angles (*27*). With increasing offset, the likelihood of collecting the backscattered signal is shifted from the purely normal to off-axis backscattering. Thus, objects that have similar normal backscattering coefficients may have vastly different angle scattering properties, which are linked to their scattering cross section.

**Fig. 5.**
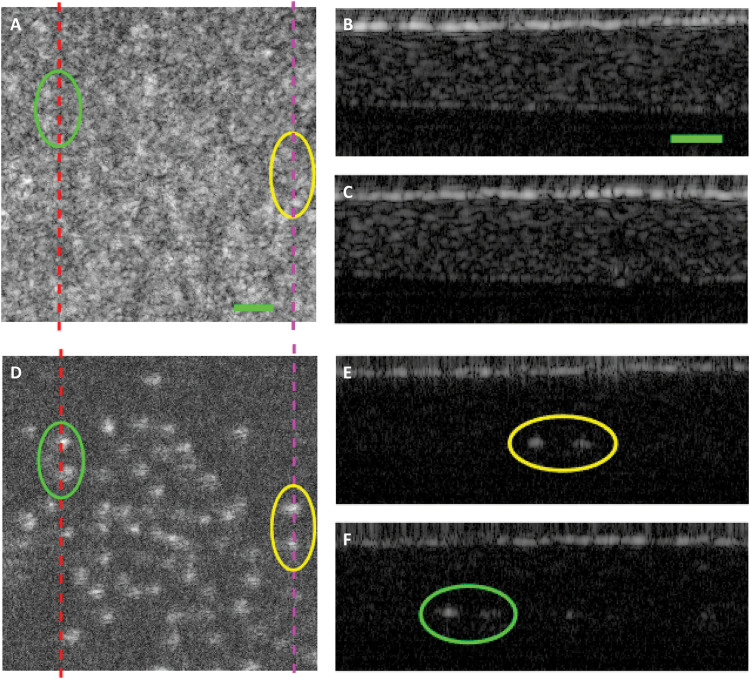
SO-OCT provides contrast based on scattering properties. (**A** to **F**) Polystyrene beads (100 μm) at a depth of 200 μm in butter, images acquired with 800-nm OCT system. (A and D) En face images with *s* = 0 μm and *s* = 80 μm, respectively, averaged more than 1.2 to 1.3 mm in optical depth. (B and C) B-scans at positions indicated by the red and purple lines in (A). (E and F) B-scans indicated by the red and purple lines in (D). The green and yellow circles indicate 100-μm beads that have been revealed from the background in SO-OCT compared with conventional OCT. Scale bars, 200 μm.

As a consequence, the contrast between structures with different scattering properties is rendered differently in SO-OCT relative to conventional OCT; structures within the range of preferential off-axis scattering, hereby termed mesoscale structures, are rendered with better contrast. We demonstrate this principle with two experiments shown in Fig. 5 and fig. S2. First, using the OCT system with central wavelength of 800 nm described in Fig. 2A, we imaged a phantom that comprised embedded polystyrene beads (100 μm in diameter) in a thick layer of butter. Most polystyrene beads are located on one plane at a depth of 200 μm below the surface. The size of the beads is much greater than the wavelength; thus, they scatter across a broad range of angles, while the butter comprises lipid molecules, which are likely to be predominantly forward and backscattering. Figure 5 (A to C) shows the conventional OCT images. The detected scattering from beads and butter is similar and nearly indistinguishable. When an offset is introduced (Fig. 5, D to F), the greater component of angular scattering possessed by the mesoscale beads clearly contrasts them in the image. In the B-scans shown in Fig. 5 (E and F), the white dots clearly show the presence of beads in the butter as the signal-to-background ratio is enhanced. For a second demonstration, using the OCT system with central wavelength of 1295 nm described in Fig. 2B, we used a similar phantom composed of 0.1% w/w Ti0_2_ microparticles with mean diameter < 5 μm uniformly dispersed in a polydimethylsiloxane (PDMS) gel (see the Supplementary Materials).

Together, these two experiments demonstrate the power of SO-OCT to reveal or enhance the contrast of hidden mesoscale structures within scattering samples. It is likely that, with an off-axis geometry, substantial contrast enhancement can be achieved in samples that have clearly separate scattering angle profiles, such as subresolution scattering media versus beads, specular reflectors, or United States Air Force (USAF) targets.

### SO-OCT imaging in soft tissue: Zebrafish

Opaque biological tissue, in particular, rapidly attenuates the OCT signal with depth, reaching the noise floor by 0.5 to 1 mm (*28*). Furthermore, it comprises a spectrum of scatterer sizes. This makes SO-OCT suitable for imaging complex biological samples.

**Fig. 6.**
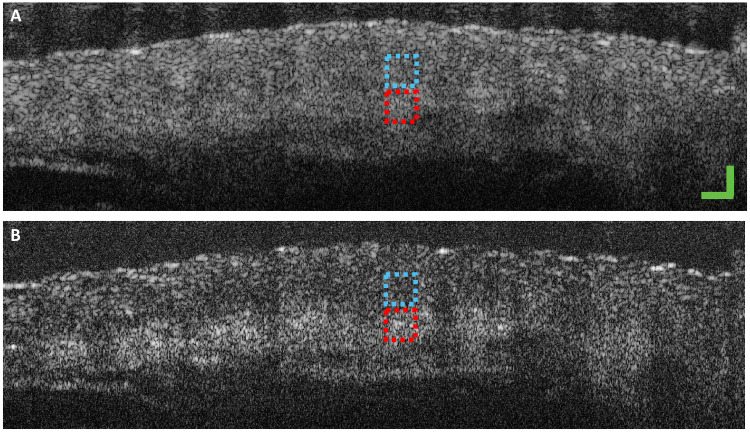
SO-OCT in zebrafish demonstrates enhanced CNR between tissue layers. B-scans of a zebrafish with (**A**) conventional OCT and (**B**) with 50-μm offset acquired with the OCT system centered at 800 nm. Red and blue boxes indicate the regions used to calculate the CNR. CNR for (A) is 0.45 dB and for (B) is 1.29 dB. Scale bars, 200 μm.

Here, we use an adult zebrafish to verify the performance of our SO-OCT system (*29*). These images were acquired using the 800-nm SO-OCT system described in Fig. 2A. The zebrafish is formalin-fixed and is placed in a petri dish with phosphate-buffered saline solution. Figure 6  shows a conventional and offset scan (*s* = 50 μm) at the same location, taken through dorsal muscle and cartilaginous spine. The SO-OCT image demonstrates improved contrast between tissue layers. To quantify this improvement, we calculated the CNR (*30*) for the regions indicated by the red box and the blue box for each B-scan. These regions were selected because they represent contrast between two different tissue types within each sample. Poor contrast between the two regions is seen in the non-offset OCT. However, enhanced contrast is attained using a spatial offset, which is likely resulting from the variations in the dominant factors determining scattering properties of the regions. Furthermore, a greater clarity of the ventral side of the spine is achieved. The spine bone structures are revealed more clearly with a greater than twofold enhancement in contrast. Another example using OCT images of an adult krill eye that shows the capability of our SO-OCT to reveal features at depth can be found in the Supplementary Materials.

**Fig. 7.**
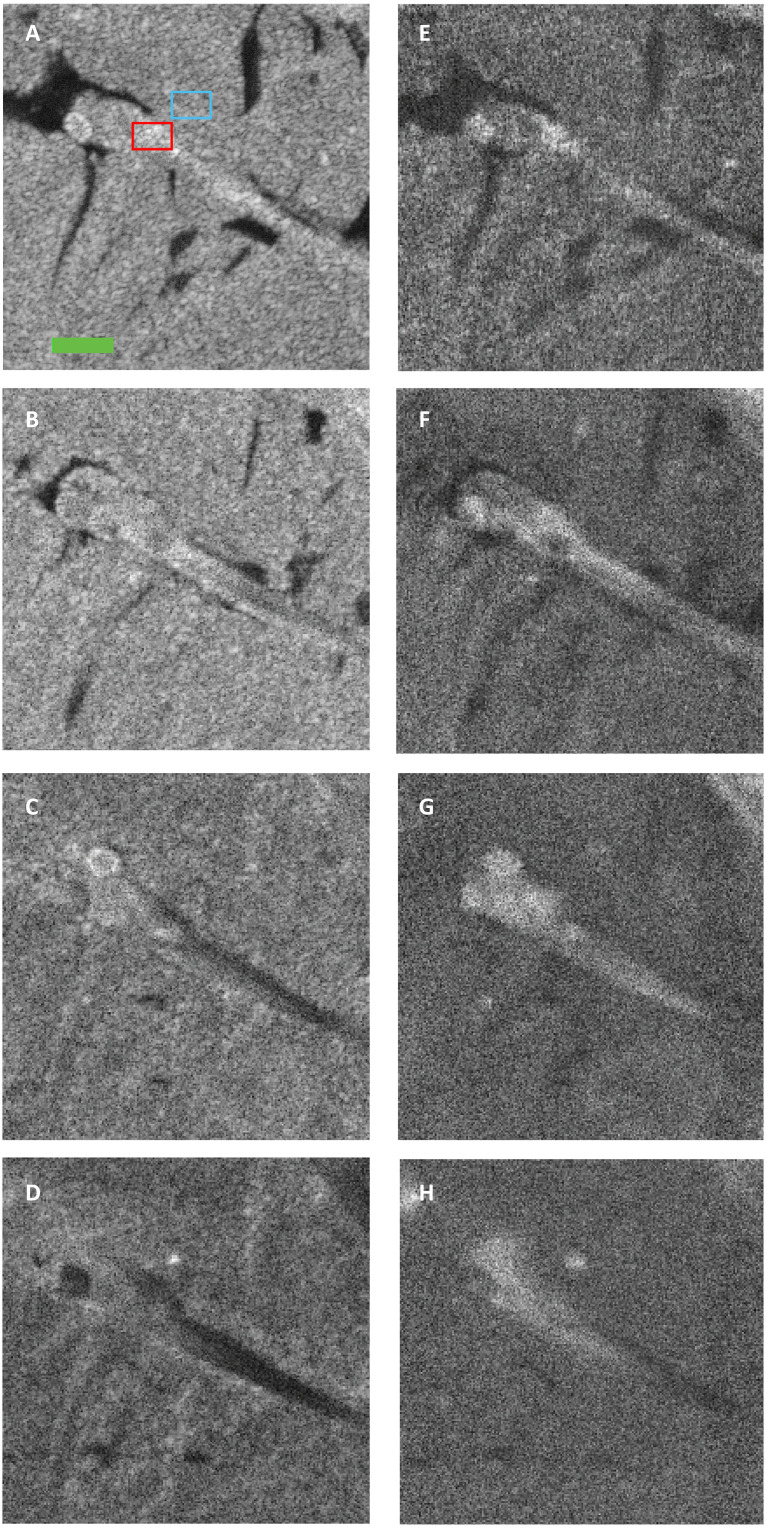
En face images of a 4-day postfertilization zebrafish embedded in scattering phantom acquired with the OCT system centered at 800 nm. (**A** to **D**) Standard OCT en face images at different depth. (**E** to **H**) SO-OCT en face images with a spatial offset of *s* = 50 μm. Figures in each row show the en face images at depths of 50, 200, 350, and 500 μm, respectively, in the scattering phantom. Red and blue rectangles indicate the regions of interest used for calculating the CNR as shown in Fig. 8. The green scale bar indicates 500 μm in length.

**Fig. 8.**
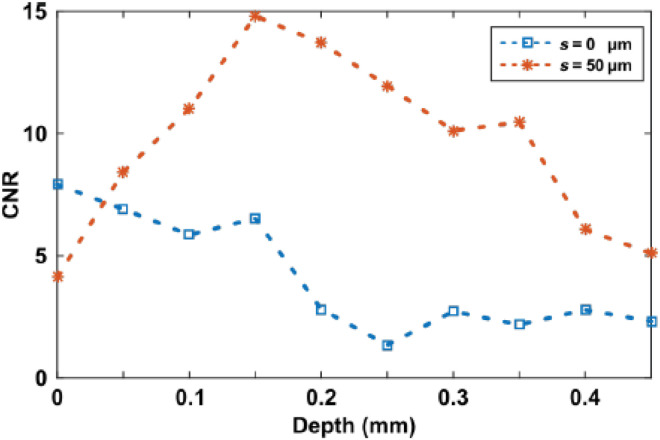
CNR as a function of the depth in zebrafish embedded in a scattering phantom. Blue and red data points show the CNR changes both with and without an offset of *s* = 50 μm.

To further show contrast improvements using the SO-OCT system, we have embedded a fixed zebrafish (4 days after fertilization) in scattering media. The media were fabricated by mixing 0.8- and 0.5-μm polystyrene beads in agarose gel. The mean free path of these scattering media *l*_s_ is estimated as 160 μm. Figure 7 shows en face images at different depths for both standard OCT and SO-OCT. Superficially, CNR in SO-OCT images is slightly lower than that in standard OCT. However, the CNR degrades at depth in standard OCT, while it is enhanced in the SO-OCT. This is validated by our theoretical framework, which demonstrates that the superficial OCT signal is dominated by ballistically scattered light that is blocked by SO-OCT. Visually, it is evident that the attenuation of the signal in the zebrafish is substantially reduced in SO-OCT. Already at 350 μm, morphological contrast in conventional OCT comprises the shadowing from surface structure, while, in SO-OCT, a greater scattering intensity to background is still seen. As shown in Fig. 8, one can achieve a maximum enhancement of 8.8 in CNR at a depth of 250 μm. Even in the deeper layers, one still can achieve over twofold minimum enhancement.

### SO-OCT imaging in hard tissue: Ex vivo mouse femur

**Fig. 9.**
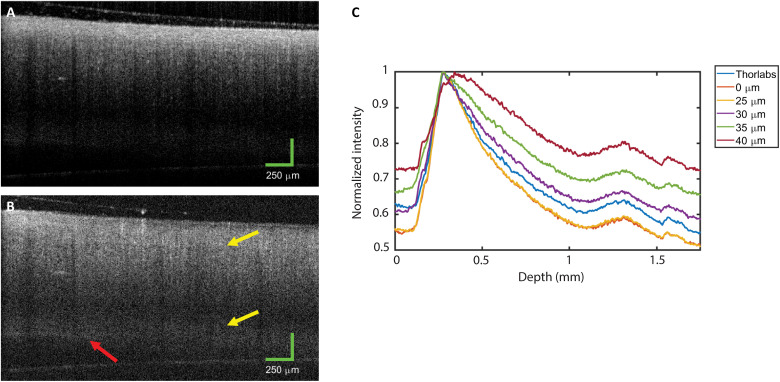
OCT B-scan images of an ex vivo mouse femur acquired with the OCT system centered at 1295 nm as shown in Fig. 2B. (**A**) Conventional OCT. (**B**) *s *= 40 μm. The SO-OCT demonstrates enhanced contrast between tissue layers. The yellow arrows indicate the boundary between the hard bone and marrow, and the red arrow indicates the bottom surface of the bone, which is revealed by the enhanced contrast in the SO-OCT image. (**C**) OCT signal intensity averaged across the lateral direction versus depth and normalized to the surface intensity at different offsets. The line labeled “Thorlabs” indicates the signal collected through the standard setup without the additional components for SO-OCT. We can clearly observe a reduction in the total effective attenuation as the offset is increased.

One of the main potential advantages of SO-OCT is the opportunity to improve CNR in highly scattering media, for example, hard tissue like bone and cartilage. Only a few studies have investigated OCT in bone because the high multiple scattering severely limits the imaging depth (*31*, *32*). For this study, we reverted to the 1295-nm OCT system described in Fig. 2B to image mouse bones to assess the opportunities for improving imaging in highly scattering tissue. Images of the bones with conventional OCT and *s* = 40 μm offset are shown in Fig. 9 (A and B), respectively. Visually, it is evident that the attenuation of the signal in the SO-OCT image is substantially less than that in the conventional OCT image. The attenuation between the top and bottom surface of the bone was reduced by more than 24 dB for an offset of *s* = 40 μm. The boundary between the hard bone and marrow is much more clearly defined, and the bottom surface of the bone is more visible. In addition, we have plotted the OCT signal intensity averaged over the lateral direction and normalized to the surface intensity in Fig. 9C. In agreement with modeling in Fig. 3D, the overall OCT signal attenuation rate is reduced as the offset is increased. This enables the dynamic range of the image to be adjusted so that the bottom surface of the bone can be better visualized. We note that we were able to achieve a similar attenuation rate through the unmodified Thorlabs system and through the add-on with no offset (*s* = 0) although some signal was lost because of the additional optical components. The signal for the offset *s* = 25 μm overlaps with the signal for *s* = 0 μm because *s* = 25 μm corresponds to an offset of less than the pinhole diameter, so some ballistically scattered light is still detected. In general, the relative intensity between the bottom and top surface of the bone is equalized as the offset is increased. This is, in part, due to the reduction of the ballistic signal from near the sample surface. A slight broadening of the peaks is consistent with the trade-off between attenuation and resolution demonstrated in Fig. 4.

## DISCUSSION

Image formation at depth remains a major limitation of OCT. There has been formidable development into extending the imaging depth through the reduction of multiple scattering using alternative optical geometries (*15*) and computational adaptive optics (*12*, *19*). Such developments have been inspired by optical microscopy, where multiple scattering scrambles microscale features beyond depths corresponding to the mean free path of the media. In OCT, however, the coherence time is substantially greater than the temporal dispersion caused by a few multiple scattering events (*20*). Thus, OCT withstands and forms images beyond 7 to 12 times the mean free path length (*12*). The more pertinent problem, which has not been given due attention, is whether signal from depth can be collected in the presence of orders-of-magnitude higher surface scattering signal—the practical dynamic range problem of frequency domain OCT with spectrometer-based detection (*18*). OCT signal is typically lost, not when structural contrast is scrambled, but when the OCT SNR reaches unity.

Using SO-OCT, we demonstrate that we can achieve greater contrast at depth while increasing the proportion of collected multiply scattered light. This is achieved by the preferential collection of multiply scattered light, counteracting conventional exponential attenuation of intensity with depth by the increasing likelihood of multiple scattering events needed to reach the offset detector aperture. Such a phenomenon may be considered as physical attenuation compensation. A similar effect may be achieved by using structured beams that increase in power with depth, such as attenuation-compensated Airy or Bessel beams demonstrated for other optical modalities (*33*). However, SO-OCT also allows the reduction of the ballistic component from close to the sample surface, which enables further optimization of the dynamic range of the detector to the signal deeper in the sample that cannot be achieved using structured beams alone.

Furthermore, because of the physical offset, a different profile of the scattering phase function (*26*) is sampled using SO-OCT. As we have demonstrated here, this may increase contrast depending on the various factors that affect the scattering properties. In particular, it enhances the contrast of mesoscale features, i.e., features much larger than the wavelength, relative to bulk scattering from structures smaller than the wavelength. In addition, by imaging at a collection of offsets, the angular distribution of the scattering function may be recovered. This may enable an alternative form of contrast in OCT or even the spatial discrimination of the mean scatterer cross section in samples. Sequences of spatially offset B-scans could also be compounded to preserve the clarity at low depth and signal strength at depth.

Because of the simple geometry used in SO-OCT, this technique can be easily combined with other approaches, such as adaptive optics (*34*) and beam shaping (*16*, *35*, *36*), in order for the illumination light to penetrate into turbid samples deeper. While this study investigated imaging at 800 and 1300 nm, we expect that the use of even longer wavelengths would also have improved imaging depth with SO-OCT (*37*).

Furthermore, we remark that, in our demonstration, greater contrast was achieved despite increasing the contribution of multiple scattering. Considering that most approaches to date have aimed to minimize multiple scattering (*12*, *15*, *19*), our results suggest that the problem of OCT imaging at depth should be distinguished from optical microscopy at depth. Our modeling indicates that multiply scattered light still contains information that contributes to the measured OCT signal. Our original geometry confirms that we should consider this issue from the perspective of attenuation, dynamic range, and the efficiency of photon collection. There is a clear link between the optimal collection geometry and the scattering properties of the sample; as such, there will be a link between the size of the features rendered with enhanced contrast and the optimal offset.

Last, we note several opportunities for future development of this technique. As we show in Fig. 3C, the offset can be tuned to optimize contrast at a particular depth in the sample. We expect that the optimal offset parameters and achievable enhancement of CNR will depend on the scattering properties of the sample. The modeling presented here also provides a platform for extracting the scattering properties directly from the OCT images. In addition, the enhancement of mesoscale structures in the image could aid in identifying different structures and boundaries between types of tissue in scattering biological samples.

## MATERIALS AND METHODS

### Experimental design

There are several factors that limit the effective penetration depth in OCT, namely, multiple scattering, sensitivity, and dynamic range. The effect of multiple scattering in OCT has been studied in much detail (*10*, *20*) and can be represented by the ratio of the collection of single-to-multiple scattered light within the coherence volume. For typical OCT systems and sample properties, multiple scattering starts to dominate beyond the mean free path (1/μ_s_; μ_s_ is the scattering coefficient) (*12*). Sensitivity in OCT is defined as the smallest reflectance that can be detected above the noise and is typically set by the shot noise limit of the detector. The OCT signal at a depth, *z*, can be represented in a simplified form for low numerical apertures (discounting focus) as (*38*)

$$\mathrm{OCT}(z)\propto P{\left[\iint_{\text{Θ}}\mu^{\prime }d\text{Θ}\right]}^{\frac{1}{2}}e^{-\mu_{\mathrm{ext}}z}$$ (2)

where *P* is related to the incident power and the detector efficiency, μ' is the angular scattering function, Θ represents the collection acceptance geometry, and μ_ext_ is the extinction coefficient. Depending on the system and sample properties, signal attenuation may reach the noise limit within 1 to 3 mm (*12*). Greater collection at depth can be achieved with greater illumination power, detector exposure time, or higher numerical apertures, which bring an additional challenge of limiting the depth of field. However, the more pertinent problem is in the cases where, at shallow depth, the extinction is small and the specular reflection from tissue is high (typically from refractive index mismatch between tissue and the environment), leading to the saturation of the detector. The high relative attenuation with depth leads to the photons collected at depth to be lost within the quantization noise of the detector. This is known as the dynamic range and is typically limited to 40 to 60 dB in spectral domain OCT (*39*). In many instances, this limit may be reached first and may even be prominent within sub-1-mm-depth ranges. This can be especially seen when refractive index matching is not used, for instance, in noncontact imaging. This may be alleviated by reducing specular reflections, for example, with dark-field illumination (*17*), or by reducing the scattering with optical clearing (*27*). An original approach to increase penetration depth is to gain control to the component collecting the backscattered light, i.e., ∬_Θ_μ'*d*Θ. We are unable to vary μ′, as it is intrinsic to any given sample; however, the angular component that we are sensitive to may be tailored by the OCT geometry. In previous works such as dark-field (*17*) and dual-axis (*15*, *16*) OCT, Θ was effectively varied to preferentially collect different ratios of specular, and Mie scattering regimes, which have added to the reason demonstrations with scattering beads and reflective targets were quite compelling.

In our approach, we recognize that, while multiple scattering is ultimately detrimental to imaging, if the temporal dispersion is below the temporal coherence of the optical source, then its effect on the image is minimal (*20*). Furthermore, multiple scattering increases with depth. By introducing a spatial offset in the collection path, the collected signal with depth is scaled with the likelihood of multiple scattering. In addition, in contrast to previous methods, the approach is able to vary Θ with depth, preferentially collecting a higher proportion of multiply scattered light with depth and completely eliminating the ballistic light. These factors lead to an effective physical means of attenuation compensation, which has been verified by numerical simulation for similar geometries in other works (*23*, *40*). Furthermore, spatial offset varies the collection of off-axis scattered light, which can further increase contrast between samples with different scattering properties. For instance, in the Mie scattering regime, particles that are much smaller than the wavelength of light exhibit greater off-axis scattering than particles on the order of the wavelength and will be detected to a greater proportion with higher offsets.

### Modeling of theoretical framework

Theoretical modeling of the physical principle of SO-OCT was performed using the EHF model (*10*, *41*). The EHF model is a wave-based model of image formation in OCT and an analytical solution to the scalar wave equation that, by its nature, can more accurately model the wave-like behavior of light in tissue including interference and diffraction effects when compared with numerical methods like Monte Carlo simulations. It is the first OCT model that adequately includes both the ballistic and multiply scattered components of the signal. In addition, the EHF model can more accurately model the impact of tissue optical properties on OCT image formation (*42*). This is particularly important for SO-OCT because the ideal imaging parameters may depend on the scattering properties of the sample.

There is ample experimental evidence that the EHF model yields a valid description of OCT image formation in scattering media in both the single and multiple scattering regimes (*10*, *43*, *44*). As such, a physical model of image formation in SO-OCT can be derived from an extension of the EHF model of a coaxial OCT system. We have already demonstrated similar extensions to the EHF model to consider the effects of multilayer scattering (*45*) and to incorporate the absorption coefficient (*44*). Building on this work, we can derive expressions for the heterodyne efficiency factor and the signal-to-noise ratio as a function of depth for SO-OCT. The full derivations are presented in the Supplementary Materials.

We acknowledge that the implementation where the illumination and collection paths are offset and parallel represents one case of a broader framework encompassing the spatial and angular offset between the illumination and collection paths. Thus, we can extend our model to also incorporate an angular offset and connect our framework to the dual-axis scheme (*15*) by incorporating a depth-dependent offset between the illumination and collection paths: *s*(*z*) = *z* tan α + *s*_0_ ≈ α*z* + *s*_0_, where α is the angular offset and *s*_0_ is the lateral offset in the plane of the tissue discontinuity (typically the focal plane). A complete derivation and discussion of the impact of angular offset can be found in the Supplementary Materials.

### Experimental implementation of SO-OCT

Two different experimental setups with central wavelengths of 800 and 1295 nm, respectively, were used in this study. Through the use of these two systems, we validate that SO-OCT is applicable over the broad parameter space where OCT is used and that the selection of the optimal wavelength can be based on the sample properties similarly to conventional OCT. As such, samples were carefully selected on the basis of the optical properties to highlight the imaging capabilities of each system. The first SO-OCT system (Fig. 2A) is entirely homebuilt and is based on a Mach-Zehnder interferometer rather than a Michelson interferometer. We chose this particular configuration because the sample arm and reference arm are completely decoupled to freely tune the spatial offset *s*. The versatility of this homebuilt system allowed us complete control to investigate the parameters that affect the optimal offset for a particular sample.

The second experimental setup is an adaptation of a commercially available frequency domain OCT system (TELESTO-II, Thorlabs Inc., Newton, NJ, USA), with a custom-built add-on enabling spatially offset detection (Fig. 2B). This implementation allowed us to demonstrate that SO-OCT can be easily adopted with existing OCT systems. Despite not being able to independently tune all parameters, we could still demonstrate improvement in CNR with the system based on the commercially available instrument. Detailed descriptions of the components used to build both setups can be found in the Supplementary Materials.

## Supplementary Materials

### This PDF file includes:

Supplementary Text Figs. S1 to S3 References
