# Supplementary material for: Spatially offset optical coherence tomography: Leveraging multiple scattering for high-contrast imaging at depth in turbid media
Source: Sci Adv. 2023 Jul 7;9(27):eadh5435. doi: 10.1126/sciadv.adh5435 (PMC10328402; doi:10.1126/sciadv.adh5435)
Supplement: Supplementary file 1 — Supplementary Text Figs. S1 to S3 References [file sciadv.adh5435_sm.pdf]

Science Advances
AAAS

Supplementary Materials for

Spatially offset optical coherence tomography: Leveraging multiple scattering for high-contrast imaging at depth in turbid media

Gavrielle R. Untracht *et al.*

Corresponding author: Peter E. Andersen, peta@dtu.dk; Kishan Dholakia, kishan.dholakia@adelaide.edu.au

Sci. Adv. **9**, eadh5435 (2023)
DOI: 10.1126/sciadv.adh5435

This PDF file includes:

Supplementary Text
Figs. S1 to S3
References

Supplementary Text

Derivation of extended Huygens-Fresnel model for image formation in spatially offset OCT

Here we derive an analytical expression for the heterodyne efficiency factor in spatially offset OCT (SO-OCT) based on the extended Huygens-Fresnel model. The proposed framework is a wave-based model which provides a complete framework for SO-OCT including both the lateral and angular offset. This derivation closely follows that presented in Thrane et al. (*10*) but modified to incorporate absorption and an offset between the illumination and collection paths in the sample arm. As a starting point, consider a conventional OCT system based on a Michelson interferometer. The light in the sample arm is incident on a discontinuity of tissue refractive index at a depth z from the tissue surface. A lens with focal length f is placed a distance d from the tissue surface such that the focal plane coincides with the tissue discontinuity. The optical path length of the reference arm is matched to the optical path length of the focal plane. The bulk tissue is characterized by the scattering coefficient, μ_s, a root mean squared (rms) scattering angle, θ_{rms}, and the absorption coefficient, μ_a. For an overview of the sample arm geometry for conventional OCT and SO-OCT, we refer to **Fig. 1a and 1b**.

Mixing the field in the sample arm backscattered at the tissue discontinuity, U_s, with the reference arm field, U_R, the mean squared heterodyne signal current at the photodetector can be expressed using the mutual coherence function, Γ, as (*19, 21*):

$$\langle i^2(z)\rangle = 2\alpha^2|g(\tau)|^2 \times Re[\iint \Gamma_S(\boldsymbol{p}_1,\boldsymbol{p}_2;z)\Gamma_R(\boldsymbol{p}_1,\boldsymbol{p}_2;z)\, d\boldsymbol{p}_1 d\boldsymbol{p}_2], \quad \text{(S1)}$$

with

$$\Gamma_{R,s}(\boldsymbol{p}_1,\boldsymbol{p}_2;z) = \langle U_{R,s}(\boldsymbol{p}_1;z)U^*_{R,s}(\boldsymbol{p}_2;z)\rangle. \quad \text{(S2)}$$

Here, α is a factor converting power to current, $\boldsymbol{p}_1$and $\boldsymbol{p}_2$ are two-dimensional vectors transverse to the optical axis, and the subscripts R and S refer to the reference and sample arms, respectively. $|g(\tau)|$ is the modulus of the normalized temporal coherence function of the source, which has a value of 1 when the sample and reference arm pathlengths are matched.

The field backscattered from the discontinuity in the sample arm can be expressed using the extended Huygens-Fresnel Green's function, $G(\boldsymbol{p},\boldsymbol{r})$, which describes the propagation of a wave through an arbitrary material with random inhomogeneities using ABCD matrix transfer formalism (19). The field in the mixing plane can be written as:

$$U_S(\boldsymbol{p};z) = \int U_B(\boldsymbol{r};z)\, G(\boldsymbol{p},\boldsymbol{r})d\boldsymbol{r} = \int U_B(\boldsymbol{r};z)G_0(\boldsymbol{p},\boldsymbol{r})e^{i\phi(\mathbf{p},\mathbf{r})}d\boldsymbol{r} \quad \text{(S3)}$$

where U_B is the backscattered field in the discontinuity plane and **r** and **p** are the transverse vectors in the mixing and discontinuity planes, respectively. The Green's function can be further decomposed into the propagation in a system without inhomogeneities, G_0, and a random phase distorting factor (caused by the inhomogeneities). We assume that the statistical properties of the incident and backscattered fields are independent and that the statistical properties of the bulk tissue and the tissue discontinuity are also independent. Substituting **Eq. S3** into **Eq. S2** and omitting the implicit z dependance for simplicity, we can write the sample arm mutual coherence function as:

$$\Gamma_s(\boldsymbol{p}_1, \boldsymbol{p}_2) = \iint \langle U_B(\boldsymbol{r}_1) U_B^*(\boldsymbol{r}_2)\rangle \langle G_0(\boldsymbol{r}_1, \boldsymbol{p}_1) G_0^*(\boldsymbol{r}_2, \boldsymbol{p}_2)\rangle \langle e^{i[\phi(\boldsymbol{p}_1) - \phi(\boldsymbol{p}_2)]}\rangle d\boldsymbol{r}_1 d\boldsymbol{r}_2. \quad \text{(S4)}$$

The first term in equation S4 leads to the mean backscattered intensity in the discontinuity plane, $\langle I_B(\boldsymbol{r})\rangle$. Assuming diffuse backscattering from the discontinuity (*10*):

$$\langle U_B(\boldsymbol{r}_1) U_B^*(\boldsymbol{r}_2)\rangle = \frac{4\pi}{k^2} \delta(\boldsymbol{r}_1 - \boldsymbol{r}_2) \langle I_B(\boldsymbol{r})\rangle, \text{ and} \quad \text{(S5)}$$

$$\langle I_B(\boldsymbol{r})\rangle = \frac{R_d P_S}{\pi} e^{-\mu_a z} \left[\frac{e^{-\mu_s z} e^{\frac{-r^2}{\omega_H^2}}}{2\omega_H^2} + \frac{(1 - e^{-\mu_s z}) e^{\frac{-r^2}{\omega_{SA}^2}}}{2\omega_{SA}^2} \right], \quad \text{(S6)}$$

were, δ is the Dirac delta function, P_s is the incident power in the sample arm, and R_d is the Fresnel reflectivity of the discontinuity. Absorption is included through the introduction of the complex refractive index, $n = n_R - i n_I$, where the imaginary part of the refractive index depends on the absorption coefficient, μ_a , and the wavevector, k: $n_I = \frac{\mu_a}{2k}$, where $k = \frac{2\pi}{\lambda_0}$ and λ_0 is the mean free-space wavelength. The first term in the brackets can be interpreted to represent the ballistically scattered light in the absence of tissue inhomogeneities while the second term represents the multiply scattered light. Here, we introduce the lateral offset in the beam, s, in order to obtain the spatially offset backscattered intensity in the discontinuity plane:

$$\langle I_B(\boldsymbol{r})\rangle = \frac{R_d P_S}{\pi} e^{-\mu_a z} \left[\frac{e^{-\mu_s z} e^{\frac{-(r-s)^2}{\omega_H^2}}}{\omega_H^2} + \frac{(1 - e^{-\mu_s z}) e^{\frac{-(r-s)^2}{\omega_{SA}^2}}}{\omega_{SA}^2} \right]. \quad \text{(S7)}$$

The quantities ω_H^2 and ω_{SA}^2 are the $1/e$ intensity radii in the absence of scattering or absorption and in the presence of both scattering and absorption, respectively. These are given by (10, 21, 44):

$$\omega_H^2 = \omega_0^2 (A - \frac{B}{f})^2 + (\frac{B}{k\omega_0})^2 \quad \text{(S8)}$$

$$\omega_{SA}^2 = (1 + \mu_a \Delta z_D)^{-1} \left[\omega_0^2 (A - \frac{B}{f})^2 + \frac{B^2}{k\omega_0} + (\frac{2B}{k\rho_0})^2 (1 + \mu_a \Delta z_N) \right]. \quad \text{(S9)}$$

In these equations, A and B are the ABCD matrix elements and ω_0 is the $1/e$ radius in the lens plane. For our geometry, $A = 1$ and $B = f + \frac{z}{n}$ (in the presence of absorption, B is complex). ρ_0 is the lateral coherence length (19, 46), and is given by:

$$\rho_0 = \sqrt{\frac{3}{\mu_s z}} \frac{\lambda}{\pi \theta_{rms}} (1 + \frac{n_R d(z)}{z}). \text{ Also,} \quad \text{(S10)}$$

$$\Delta z_N = \frac{z(\omega_0^2 + \frac{\rho_0^2}{2})}{4 n_R^2 B^2} \quad \text{(S11)}$$

$$\Delta z_D = \frac{z}{2n_R^2} \left[(\frac{\omega_0}{f})^2 + (\frac{1}{k\omega_0})^2 + (\frac{2}{k\rho_0})^2 \right]. \quad \text{(S12)}$$

The dependance of d on z in **Eq. S10** is indicated to include OCT systems that employ dynamic focusing, where $d = f - \frac{z}{n_R}$.

The second term in **Eq. S4** is the Huygens-Fresnel Green's function describing propagation from the discontinuity plane to the mixing plane in the absence of tissue inhomogeneities. The Green's function will differ from that presented in Thrane et al. (*10*) due to the incorporation of absorption. With this change, the Green's function becomes (*47*):

$$G_0(\boldsymbol{p}, \boldsymbol{r}) = \frac{-ik}{2\pi B} e^{-ikL_0} e^{\frac{-ik}{2B}(Ap^2 - 2\boldsymbol{p} \cdot \boldsymbol{r} + \boldsymbol{D}\boldsymbol{r}^2)}, \tag{S13}$$

where, L_0 is the total optical path length from the discontinuity plane to the mixing plane, and A, B, and D are elements of the ABCD transfer matrix. For our geometry, $A = D = 1$ and $B = d + \frac{z}{n}$.

The third term in **Eq. S4** represents the impact of tissue inhomogeneities on wavefronts propagating through the tissue, and can be expressed as the mutual coherence function, Γ_{pt} of a point source located at the discontinuity plane and observed in the lens plane (*10*):

$$\Gamma_{pt} = \langle e^{i[\phi(\boldsymbol{p}_1) - \phi(\boldsymbol{p}_2)]} \rangle \approx e^{-\sigma^2(1 - b_\phi(\rho))} = e^{-\mu_s z} + (1 - e^{-\mu_s z}) e^{\frac{-\rho^2 \mu_s z}{\rho_\phi^2}}. \tag{S14}$$

Finally, the electric field in the reference arm (ignoring complex terms that cancel when the coherence function is calculated) can be written as:

$$U_R(\boldsymbol{p}) = \sqrt{\frac{P_R}{\pi\omega_0^2}} e^{\frac{-(p-s)^2}{2\omega_0^2}}. \tag{S15}$$

where P_R is the incident power in the reference arm. The presence of the offset, s, in the reference arm depends on the geometry of the OCT system. If the offset in the sample arm can be adjusted without impacting the reference arm, such as the 800 nm system demonstrated in this study, the offset in the reference arm can be set to 0. We include it here for generality since it will often be relevant for Michelson-interferometer-based SO-OCT.

Substituting the relevant equations into **Eq. S1** and performing the integrations over $\boldsymbol{p}$ and $\boldsymbol{r}$, we now obtain an expression for the mean square heterodyne signal current:

$$\langle i^2(z) \rangle = \langle i^2 \rangle_0 \Psi(z, s), \tag{S16}$$

where $\langle i^2 \rangle_0 = \frac{\alpha^2 P_R P_S \sigma_b}{\pi \omega_H^2}$ is the mean square heterodyne current in the absence of scattering and absorption, and $\Psi(z, s)$ is the heterodyne efficiency factor. $\Psi(z, s)$ represents the tissue attenuation due to absorption and scattering, and can be expressed as:

$$\Psi(z,s) = e^{-2\mu_a z}\left[e^{-2\mu_s z}e^{\frac{-s^2}{2\omega_H^2}} + \frac{4e^{-\mu_s z}(1-e^{-\mu_s z})}{(1+\mu_a\Delta z_D)(1+\frac{\omega_{SA}^2}{\omega_H^2})}e^{\frac{-s^2}{\omega_H^2+\omega_{SA}^2}} + \frac{(1-e^{-\mu_s z})^2\omega_H^2}{(1+\mu_a\Delta z_D)\omega_{SA}^2}e^{\frac{-s^2}{2\omega_{SA}^2}}\right], \quad \text{(S17)}$$

Extension of EHF modelling framework to encompass angular offsets

We acknowledge that the implementation where the illumination and collection paths are offset and parallel represents one case of a broader framework encompassing the spatial and angular offset between the illumination and collection paths. Thus, we can extend our model to also incorporate an angular offset and connect our framework to the dual axis scheme (*15*). For an overview of the sample arm geometry incorporating angular offset with and without a lateral offset, we refer to **Fig. 1c and 1d**.

Since no integrations are performed along the axial depth, z, **Eq. S17** can easily be generalized to encompass an angular offset α between the illumination and collection paths by incorporating a depth-dependent variable offset. The depth dependent offset can be expressed as $s(z) = z\tan\alpha + s_0$, where s_0 is the lateral offset in the discontinuity plane. In the limit of the paraxial approximation, $s(z) = z\alpha + s_0$. Thus, the final expression for the heterodyne efficiency factor incorporating both lateral and angular offset can be expressed as:

$$\Psi(z,s_0,\alpha) = e^{-2\mu_a z}\left[e^{-2\mu_s z}e^{\frac{-(z\alpha+s_0)^2}{2\omega_H^2}} + \frac{4e^{-\mu_s z}(1-e^{-\mu_s z})}{(1+\mu_a\Delta z_D)(1+\frac{\omega_{SA}^2}{\omega_H^2})}e^{\frac{-(z\alpha+s_0)^2}{\omega_H^2+\omega_{SA}^2}} + \frac{(1-e^{-\mu_s z})^2\omega_H^2}{(1+\mu_a\Delta z_D)\omega_{SA}^2}e^{\frac{-(z\alpha+s_0)^2}{2\omega_{SA}^2}}\right]. \quad \text{(S18)}$$

Building on the work presented in (*40*), we note a key difference between spatially-offset OCT and dual-axis OCT. When $\alpha \neq 0$, some ballistic light scattered at a particular angular range is collected by the detector. Indeed, this configuration selectively collects ballistically scattered light from the elliptical region where the illumination and collection paths overlap. Conversely, if $\alpha = 0$ and s is greater than the lateral resolution, all ballistic light is rejected. The angular relation between the illumination and collection paths in dual-axis OCT is designed to selectively collect light scattered ballistically at a particular angle to the illumination beam and is based on the principle that multiply scattered light, while collected by the coherence gate, contributes only an unwanted background signal that results on the degradation of OCT image contrast. The impact of angular offset on the heterodyne efficiency factor (and thereby the OCT signal intensity) is shown in **Fig. S1.** Conversely, recent work modelling the OCT signal using the extended Huygens-Fresnel

model has shown that multiply scattered light does indeed contain information about sample structure (*10*). As such, the spatial offset allows *selective* collection of the multiply scattered light, which enables deeper imaging since the multiply scattered light has typically travelled deeper into the sample. The relative dynamic range of images with lateral and angular offset can be seen by comparing the slopes of the lines plotted in **Fig. 2d and S1a**; a shallower slope indicates that a smaller detector dynamic range is required to visualize the signal. Note that in **Fig. S1a**, the normalized contrast would be equivalent to the heterodyne efficiency factor since the peak contrast is always at the sample surface. The optimal configuration including the lateral and angular offset must be determined based on the specific geometry and scattering properties of the sample. Since the offset can easily be tuned from $s = 0$ to various offsets with $s > 0$, images with and without the ballistic component can be acquired using the same OCT system. The magnitude of the spatial offset and the angular offset represent two factors that can be independently tuned based on the sample scattering properties in order to optimize the contrast at different regions by controlling the ratio of ballistically and multiply scattered light that are collected from different regions of the sample.

Enhanced contrast in microbead phantoms: microparticles in PDMS gel

In order to further explore the contrast dependance on the angle-dependent scattering properties of the sample, we performed a second experiment to demonstrate how the relative contrast between different sized structures changes with increased offset. Using the OCT system with central wavelength of 1295 nm described in **Fig. 2b** we employed a phantom comprised of 0.1% w/w $Ti0_2$ microparticles with mean diameter < 5 µm uniformly dispersed in a PDMS gel. A ~1mm slab of this phantom was placed on top of a flat piece of transparent epoxy. It is clear from **Figs. S2a-d** that the bulk scattering contrast of the $Ti0_2$ microparticles, which fall within the predominantly forward and backscattering regime, is greatly *reduced* as the offset is *increased*, while the contrast of the bottom layer of the phantom relative to the bulk scattering is *increased*. To quantify this improvement, we calculated the contrast-to-noise ratio (CNR) (*30*) for the depth range between the blue lines (signal) and the depth range between the red lines (background "noise") for each B-scan. The full image width over the indicated depth range was used to calculate the CNR. The red region was selected to show a region of the image where the intensity of the smaller particles is reduced relative to the larger surface, indicated by the blue region. Here CNR is defined as $|\mu - \mu_b|/\sqrt{\delta^2 + \delta_b^2}$, where μ, δ, μ_b, and δ_b denote the mean and standard deviation of the pixel intensities in the signal region and background region, respectively. We measured values of 1.01 dB, 1.29 dB, 1.43 dB, and 1.47 dB for offsets of s = 0, 30, 40, 50 µm, respectively, which demonstrates that the CNR at depth increases as the offset increases.

OCT imaging in soft tissue: krill eyes

We take OCT images from an adult krill eye to show the capability of our SO-OCT system to reveal features at depth (*39*). **Figs. 6 c and d** show the non-offset OCT and SO-OCT B-scans (s = 150 µm), respectively, on the eye of an adult krill. The top surface of the eye is well-defined in both B scans. However, the bottom surface of the eye is poorly defined because of rapid signal attenuation. The signals from the bottom surface can be enhanced when an offset, in this case of s = 150 µm, is applied. As shown in our modelling (**Fig. 3**), the selected offset specifies a depth in the image which has optimal contrast enhancement. Thus, the large offset employed here was selected to demonstrate an improved contrast deeper in the sample, at the depth corresponding to the bottom surface of the eye. A higher CNR value of 5.93 dB was obtained in SO-OCT

compared to 3.35 dB in conventional OCT, which corresponds to an enhancement factor of 1.77 in the obtained image quality.

Detailed descriptions of experimental setups for SO-OCT

The first SO-OCT system (**Fig. 2A**) is entirely home built and is based on a Mach-Zehnder interferometer. In this configuration, we use a superluminescent diode (SLD) laser (S850, Superlum, Cork, Ireland) with central wavelength of 800 nm and a bandwidth of 14 nm as our light source, which is split into two paths by a beamsplitter B1, 90%, into the sample arm and 10% into the reference arm. A low NA microscope objective (LSM03-BB, Thorlabs, Newton, NJ, USA) is used for focusing the light up to 1 mm into the sample. The focus plane is imaged on a CCD camera (CCD1), which is on the direction of zeroth diffraction order of the grating (GR1). The first diffraction order is focused onto a line CCD camera (CCD2), which provides the spectra information. The reference arm propagates through the same optical path except the dispersion compensation (DC) before it interferes with the sample arm. As indicated by the double arrow in **Fig. 2A**, the tuning of the spatial offset *s* is realized by simply shifting the lens L2 using a translation stage. The linear relationship between the shift of L2 and the spatial offset *s* is calibrated carefully in advance by measuring the laser spot on CCD1. In this way, it is straightforward to acquire OCT images with or without spatial offset in order to compare the performance. The exposure time of the camera was adjusted to ensure comparable dynamic range in all images.

The second experimental setup is an adaptation of a commercially available frequency domain OCT system (TELESTO-II, Thorlabs Inc., Newton, NJ, USA), with a custom-built add-on enabling spatially offset detection (**Fig. 2B**). This system is based on a Michelson interferometer and uses an SLD with a central wavelength of 1295 nm and a bandwidth of 217 nm. A circulator (CIR) was added in between the light source and the interferometer. A 50/50 beamsplitter (BS) splits the light into the sample and reference arms. A low NA microscope objective (LSM03, Thorlabs Inc., Newton, NJ, USA) with nominal resolution of 13 μm in air was used to focus light onto the sample. A pinhole on a translation stage was introduced in the collection path in order to tune the offset, as indicated by the arrows in **Fig. 2B**. One difference compared with the 800 nm setup is that, by placing the pinhole along the collection path, an offset is also introduced to the light collected from the reference arm. Thus, the power in the reference arm must be increased to compensate as the offset is increased. This could impact the sensitivity of the measurement especially for large offsets when the maximum reference arm power has been reached. All images were acquired in high sensitivity mode with an A-scan rate of 5.5 kHz.

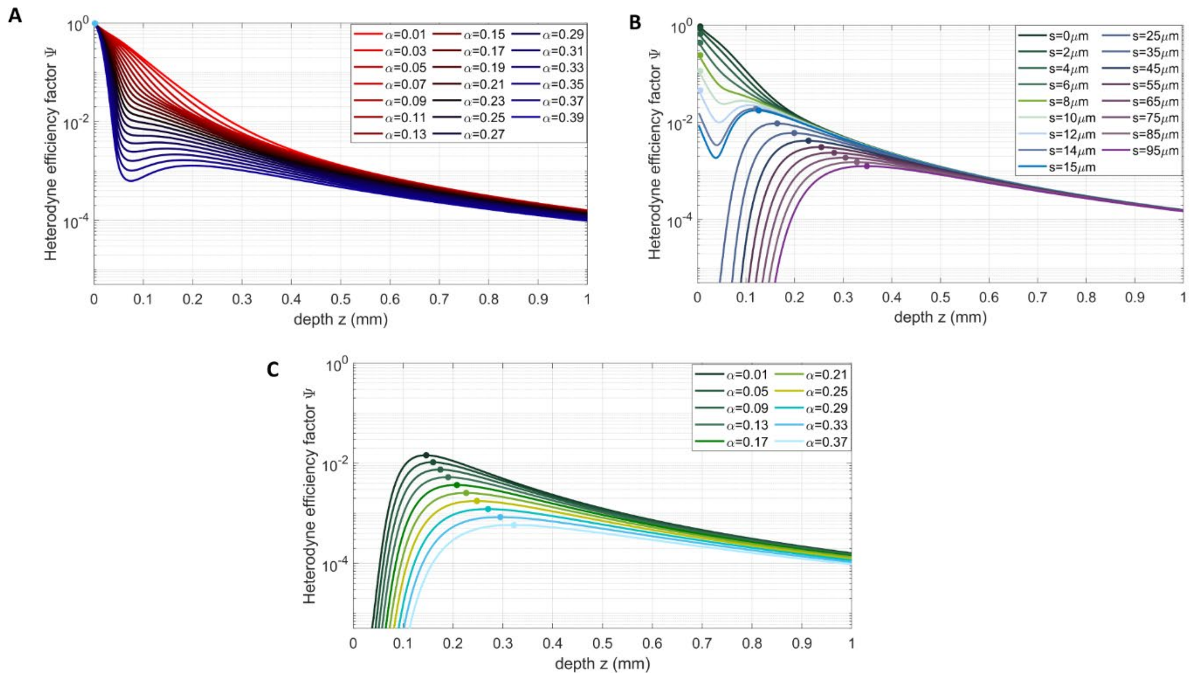

Fig. S1.

Calculations of the heterodyne efficiency factor (Ψ) demonstrating the impact of the lateral and angular offset. (a) is the heterodyne efficiency factor with $\boldsymbol{s_0 = 0}$ and various angular offsets. The blue dot in the upper left corner indicated the depth with highest contrast for all angular offsets. (b) shows the heterodyne efficiency factor with $\boldsymbol{\alpha}$ = 0.6 and various lateral offsets. The circles plotted on each line indicate the depth with maximum contrast. (c) shows the heterodyne efficiency factor for $\boldsymbol{s_0 = 25}$ for various angular offsets. The circles plotted on each line indicate the depth with highest contrast. The following system parameters representing the system described in Fig. S4 were used for these calculations: $\boldsymbol{\lambda_0 = 1295\,nm}$; $\boldsymbol{\omega_0 = 1.5\,mm}$; $\boldsymbol{f = 36\,mm}$; $\boldsymbol{n = 1.4}$; $\boldsymbol{\mu_s = 10\,mm^{-1}}$; $\boldsymbol{\mu_a = 0\,mm^{-1}}$; $\boldsymbol{\theta_{rms} = 0.3}$. All angles are in units of radians. Note that the calculations here all consider an OCT system with dynamic focusing.

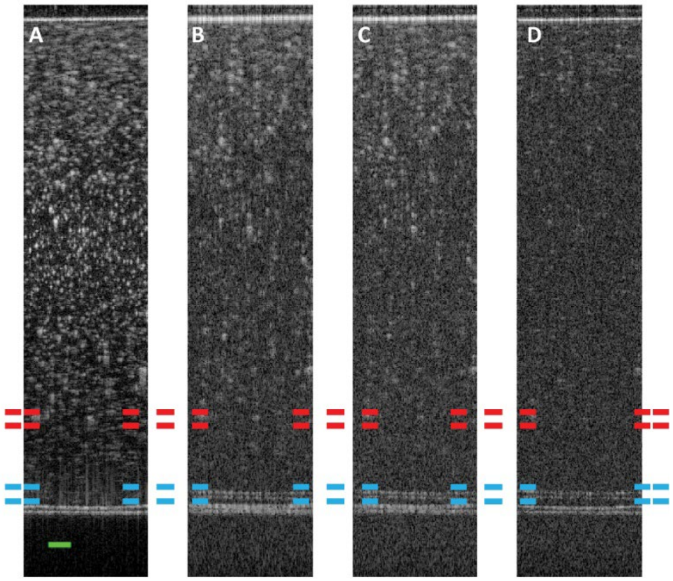

Fig. S2.

SO-OCT provides contrast based on scattering properties (second example). TiO_2 microparticles with mean diameter < 5 µm embedded in a PDMS gel, acquired with the 1295 nm OCT system, for offsets at *s* = 0, 30, 40, 50 µm (A-D, respectively). Blue and red lines indicate the depth ranges used to calculate the CNR. Scale bar indicates 200 µm.

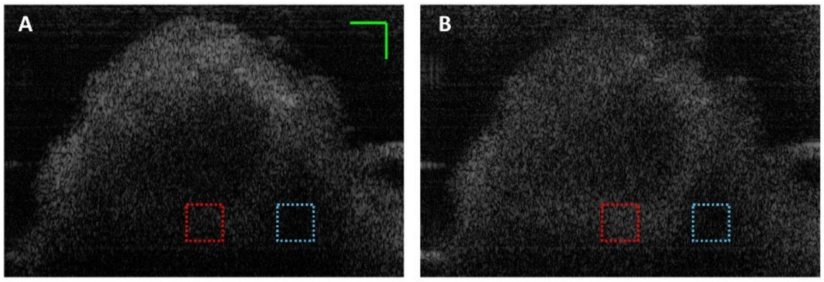

Fig. S3.

SO-OCT demonstrates enhanced contrast-to-noise ratio (CNR) deep in scattering tissue. (A) and (B) show B-scans from the eye of an adult krill with no offset and offset s = 150 µm, respectively. Red and blue boxes indicate the regions used to calculate the CNR. CNR for (A) is 3.35 dB and for (B) is 5.93 dB. Scale bars indicate 200 µm.

REFERENCES AND NOTES

1. D. Huang, E. A. Swanson, C. P. Lin, J. S. Schuman, W. G. Stinson, W. Chang, M. R. Hee, T. Flotte, K. Gregory, C. A. Puliafito, J. G. Fujimoto, Optical coherence tomography. *Science* **254**, 1178–1181 (1991).

2. J. G. Fujimoto, M. E. Brezinski, G. J. Tearney, S. A. Boppart, B. Bouma, M. R. Hee, J. F. Southern, E. A. Swanson, Optical biopsy and imaging using optical coherence tomography. *Nat. Med.* **1**, 970–972 (1995).

3. W. Drexler, U. Morgner, R. K. Ghanta, F. X. Kärtner, J. S. Schuman, J. G. Fujimoto, Ultrahigh-resolution ophthalmic optical coherence tomography. *Nat. Med.* **7**, 502–507 (2001).

4. M. C. Pierce, J. Strasswimmer, B. H. Park, B. Cense, J. F. De Boer, Advances in optical coherence tomography imaging for dermatology. *J. Invest. Dermatol.* **123**, 458–463 (2004).

5. F. Alfonso, M. Paulo, N. Gonzalo, J. Dutary, P. Jimenez-Quevedo, V. Lennie, J. Escaned, C. Bauelos, R. Hernandez, C. MacAya, Diagnosis of spontaneous coronary artery dissection by optical coherence tomography. *J. Am. Coll. Cardiol.* **59**, 1073–1079 (2012).

6. J. Wang, Y. Xu, S. A. Boppart, Review of optical coherence tomography in oncology. *J. Biomed. Opt.* **22**, 121711 (2017).

7. P. Targowski, B. Rouba, M. Góra, L. Tymińska-Widmer, J. Marczak, A. Kowalczyk, Optical coherence tomography in art diagnostics and restoration. *Appl. Phys. A* **92**, 1–9 (2008).

8. D. Levitz, M. T. Hinds, A. Ardeshiri, S. R. Hanson, S. L. Jacques, Non-destructive label-free monitoring of collagen gel remodeling using optical coherence tomography. *Biomaterials* **31**, 8210–8217 (2010).

9. S. Ishida, N. Nishizawa, Quantitative comparison of contrast and imaging depth of ultrahigh-resolution optical coherence tomography images in 800–1700 nm wavelength region. *Biomed. Opt. Express* **3**, 282–294 (2012).

10. L. Thrane, H. T. Yura, P. E. Andersen, Analysis of optical coherence tomography systems based on the extended Huygens–Fresnel principle. *J. Opt. Soc. Am. A* **17**, 484–490 (2000).

11. B. Karamata, P. Lambelet, M. Laubscher, M. Leutenegger, S. Bourquin, T. Lasser, Multiple scattering in optical coherence tomography. I. Investigation and modeling. *J. Opt. Soc. Am. A* **22**, 1369–1379 (2005).

12. A. Badon, D. Li, G. Lerosey, A. C. Boccara, M. Fink, A. Aubry, Smart optical coherence tomography for ultra-deep imaging through highly scattering media. *Sci. Adv.* **2**, e1600370 (2016).

13. S. Kang, S. Jeong, W. Choi, H. Ko, T. D. Yang, J. H. Joo, J. S. Lee, Y. S. Lim, Q. H. Park, W. Choi, Imaging deep within a scattering medium using collective accumulation of single-scattered waves. *Nat. Photonics* **9**, 253–258 (2015).

14. L. K. Wong, M. J. Mandella, G. S. Kino, T. D. Wang, Improved rejection of multiply scattered photons in confocal microscopy using dual-axes architecture. *Opt. Lett.* **32**, 1674–1676 (2007).

15. Y. Zhao, W. J. Eldridge, J. R. Maher, S. Kim, M. Crose, M. Ibrahim, H. Levinson, A. Wax, Dual-axis optical coherence tomography for deep tissue imaging. *Opt. Lett.* **42**, 2302–2305 (2017).

16. E. T. Jelly, Y. Zhao, K. K. Chu, H. Price, M. Crose, Z. A. Steelman, A. Wax, Deep imaging with 1.3 µm dual-axis optical coherence tomography and an enhanced depth of focus. *Biomed. Opt. Express* **12**, 7689–7702 (2021).

17. M. Villiger, C. Pache, T. Lasser, Dark-field optical coherence microscopy. *Opt. Lett.* **35**, 3489–3491 (2010).

18. A. F. Fercher, W. Drexler, C. K. Hitzenberger, T. Lasser, Optical coherence tomography - Principles and applications. *Rep. Prog. Phys.* **66**, 239–303 (2003).

19. S. Liu, M. R. E. Lamont, J. A. Mulligan, S. G. Adie, Aberration-diverse optical coherence tomography for suppression of multiple scattering and speckle. *Biomed. Opt. Express* **9**, 4919 (2018).

20. J. M. Schmitt, A. Knüttel, Model of optical coherence tomography of heterogeneous tissue. *J. Opt. Soc. Am. A* **14**, 1231 (1997).

21. M. Chen, J. Mas, K. Dholakia, Spatially-offset optical coherence tomography, in *Conference on Lasers and Electro-Optics, OSA Technical Digest (online)* (2018).

22. H. T. Yura, Signal-to-noise ratio of heterodyne lidar systems in the presence of atmospheric turbulence. *Opt. Acta (Lond)* **26**, 627–644 (1979).

23. M. Cua, B. Blochet, C. Yang, Speckle-resolved optical coherence tomography for mesoscopic imaging within scattering media. *Biomed. Opt. Express* **13**, 2068–2081 (2022).

24. W. Xu, H. Wang, Using beam-offset optical coherence tomography to reconstruct backscattered photon profiles in scattering media. *Biomed. Opt. Express* **13**, 6124–6135 (2022).

25. M. J. Yadlowsky, J. M. Schmitt, R. F. Bonner, Multiple scattering in optical coherence microscopy. *Appl. Opt.* **34**, 5699–5707 (1995).

26. J. Piskozub, D. McKee, Effective scattering phase functions for the multiple scattering regime. *Opt. Express* **19**, 4786–4794 (2011).

27. V. V. Tuchin, *Tissue Optics: Light Scattering Methods and Instruments for Medical Diagnosis* (SPIE, ed. 3, 2015).

28. R. A. Leitgeb, W. Drexler, A. Unterhuber, B. Hermann, T. Bajraszewski, T. Le, A. Stingl, A. F. Fercher, Ultrahigh resolution Fourier domain optical coherence tomography. *Opt. Express* **12**, 2156 (2004).

29. G. J. Lieschke, P. D. Currie, Animal models of human disease: Zebrafish swim into view. *Nat. Rev. Genet.* **8**, 353–367 (2007).

30. F. Timischl, The contrast-to-noise ratio for image quality evaluation in scanning electron microscopy. *Scanning* **37**, 54–62 (2015).

31. N. Ugryumova, J. Stevens-Smith, A. Scutt, S. J. Matcher, Local variations in bone mineral density: A comparison of OCT versus x-ray micro-CT. *Coherence Domain Opt. Methods Opt. Coherence Tomogr. Biomed. XII* **6847**, 684725 (2008).

32. C. Kasseck, M. Kratz, A. Torcasio, N. C. Gerhardt, G. H. van Lenthe, T. Gambichler, K. Hoffmann, D. B. Jones, M. R. Hofmann, Comparison of optical coherence tomography, microcomputed tomography, and histology at a three-dimensionally imaged trabecular bone sample. *J. Biomed. Opt.* **15**, 046019 (2010).

33. J. Nylk, K. McCluskey, M. A. Preciado, M. Mazilu, Z. Yang, F. J. Gunn-Moore, S. Aggarwal, J. A. Tello, D. E. K. Ferrier, K. Dholakia, Light-sheet microscopy with attenuation-compensated propagation-invariant beams. *Sci. Adv.* **4**, eaar4817 (2018).

34. M. Pircher, R. J. Zawadzki, Review of adaptive optics OCT (AO-OCT): Principles and applications for retinal imaging [Invited]. *Biomed. Opt. Express* **8**, 2536–2562 (2017).

35. D. Lorenser, C. Christian Singe, A. Curatolo, D. D. Sampson, Energy-efficient low-Fresnel-number Bessel beams and their application in optical coherence tomography. *Opt. Lett.* **39**, 548–551 (2014).

36. A. Curatolo, P. R. T. Munro, D. Lorenser, P. Sreekumar, C. C. Singe, B. F. Kennedy, D. D. Sampson, Quantifying the influence of Bessel beams on image quality in optical coherence tomography. *Sci. Rep.* **6**, 23483 (2016).

37. V. M. Kodach, J. Kalkman, D. J. Faber, T. G. van Leeuwen, Quantitative comparison of the OCT imaging depth at 1300 nm and 1600 nm. *Biomed. Opt. Express* **1**, 176–185 (2010).

38. J. Xi, Y. Chen, X. Li, Characterizing optical properties of nano contrast agents by using cross-referencing OCT imaging. *Biomed. Opt. Express* **4**, 842–851 (2013).

39. N. Bellini, M. J. Cox, D. J. Harper, S. R. Stott, P. C. Ashok, K. Dholakia, S. Kawaguchi, R. King, T. Horton, C. T. A. Brown, The application of optical coherence tomography to image subsurface tissue structure of antarctic krill Euphausia Superba. *PLOS ONE* **9**, e110367 (2014).

40. Y. Zhao, K. K. Chu, E. T. Jelly, A. Wax, Origin of improved depth penetration in dual-axis optical coherence tomography: A Monte Carlo study. *J. Biophotonics* **12**, e201800383 (2019).

41. H. T. Yura, S. G. Hanson, Optical beam wave propagation through complex optical systems. *J. Opt. Soc. Am. A* **4**, 1931 (1987).

42. P. Gong, M. Almasian, G. van Soest, D. M. de Bruin, T. G. van Leeuwen, D. D. Sampson, D. J. Faber, Parametric imaging of attenuation by optical coherence tomography: Review of models, methods, and clinical translation. *J. Biomed. Opt.* **25**, 1–34 (2020).

43. V. D. Nguyen, D. J. Faber, E. van der Pol, T. G. van Leeuwen, J. Kalkman, Dependent and multiple scattering in transmission and backscattering optical coherence tomography. *Opt. Express* **21**, 29145–29156 (2013).

44. Z. Turani, E. Fatemizadeh, T. Blumetti, S. Daveluy, A. F. Moraes, W. Chen, D. Mehregan, P. E. Andersen, M. Nasiriavanaki, Optical Radiomic Signatures Derived from Optical Coherence Tomography Images Improve Identification of Melanoma. *Cancer Res.* **79**, 2021–2030 (2019).

45. L. Thrane, M. H. Frosz, T. M. Jørgensen, A. Tycho, H. T. Yura, P. E. Andersen, Extraction of optical scattering parameters and attenuation compensation in optical coherence tomography images of multilayered tissue structures. *Opt. Lett.* **29**, 1641–1643 (2004).

46. R. F. Lutomirski, Atmospheric degradation of electrooptical system performance. *Appl. Optics* **17**, 3915–3921 (1978).

47. A. E. Siegman, *Lasers* (University Science Books, 1986).
